# Zeolite Membrane: From Microstructure to Separation Performance

**DOI:** 10.3390/membranes12020176

**Published:** 2022-02-02

**Authors:** Tomohiro Kyotani, Hannes Richter

**Affiliations:** 1 Mitsubishi Chemical Corporation, Fukuoka R&D Center, Kurosaki-Shiroishi 1-1, Yahatanishi-ku, Kitakyushu-shi 806-0004, Japan; 2 Fraunhofer Institute for Ceramic Technologies and Systems IKTS, Michael-Faraday-Str. 1, 07629 Hermsdorf, Germany; hannes.richter@ikts.fraunhofer.de

Zeolite membrane have been investigated all over the world as an attractive tool in the development of separation processes for both liquid and gaseous components. The separation process targets dehydration, organic–organic separation, gas separation, and so on. The microstructure of the membrane plays an important role in the separation mechanism; therefore, it is very important to quantitatively understand the relationship between the microstructure and separation performance. The microstructure is determined by the distribution of zeolite crystals and grain boundaries, pore connectivity inside zeolite crystals, the mixing state of the amorphous phase, and an interface structure between the membrane and its support material, etc. This structure was characterized not only by conventional analytical methods such as X-ray Diffraction (XRD), Scanning Electron Microscopy (SEM), Transmission Electron Microscopy (TEM), Energy Dispersive X-ray Spectrometry (EDX), Infrared Spectroscopy (IR), Nuclear Magnetic Resonance (NMR) and permporometry, but also by computational molecular simulation studies. This separation technique includes pervaporation, vapor permeation and gas permeation processes. The chemical engineering analysis of the separation performance and improvements to the equipment applied to the separation processes are also important for discussing the microstructure. This Special Issue contains ten papers on experimental and computational molecular simulation studies, using various zeolites such as LTA, CHA, FAU, MFI (including silicalite-1) and ZSM-22.

Two papers explored the basic characteristics of liquid separation. Hasegawa et al. [1] reported the pervaporative dehydration performances of various organic solvents using a high-silica, CHA-type zeolite membrane at 303–373 K. The dehydration performances of the membrane were categorized to the following three types: (1) 2-propanol, acetone, tetrahydrofuran (THF) and methyl ethyl ketone (MEK); (2) ethanol and acetic acid; and (3) methanol, N,N-dimethylformamide (DMF), dimethyl sulfoxide (DMSO) and N-methyl-2-pyrolidone (NMP). The category (1) showed high permeation fluxes and separation factors because of the preferential adsorption of water due to molecular sieving. Hasegawa et al. [2] investigated the lower dehydration performances of an NaA-type zeolite membrane for high-boiling solvents such as NMP solutions, as opposed to alcohols and low-boiling solvents. This study suggested that the lower dehydration performances for the high-boiling solvents were attributed to the lower vapor pressures of water and the higher permeances of those solvents.

Four papers explored the basic characteristics of gas separation. Hasegawa et al. [3] reported the permeation properties of various gaseous components, such as H_2_, CO_2_, N_2_, CH_4_, n-C_4_H_10_, and SF_6_ of the polycrystalline CHA-type zeolite membrane with Si/Al = 18. The N_2_/SF_6_ and CO_2_/CH_4_ selectivities were as high as 710 and 240, respectively. However, the CO_2_/N_2_ selectivity was only 25. These results propose that the high-silica, CHA-type zeolite membrane is suitable for the separation of CO_2_ from CH_4_ by the effect of molecular sieving. Inami et al. [4] reported the use of a zeolite membrane that was selectively permeable to ammonia as a sensor in a sewer system. LTA-, MFI-, and FAU-type zeolite membranes were prepared and the permeation and separation performances were determined for the ternary mixture of NH_3_, H_2_S, and N_2_. The FAU-type zeolite membrane with Si/Al = 1.35 showed a high enough NH_3_ permeance and a NH_3_/N_2_ separation factor. Furthermore, the membrane modifications with silane-coupling agents and varying membrane compositions were carried out to reduce the H_2_S permeance. Hasegawa and Abe [5] reported molecular simulation studies to predict the adsorption and diffusion behaviors of CO_2_ and CH_4_ in all-silica zeolites. They investigated the novel, non-bonding interaction parameters of all-silica zeolites for the prediction of the adsorption and diffusion behaviors by focusing on the Si atom of zeolite frameworks; the adsorption isotherms of CO_2_ and CH_4_ on several zeolites could be predicted with a high accuracy. Wotzka et al. [6] presented the applicability of MFI membranes for H_2_O/CO_2_ separation by means of realistic adsorption isotherms computed by configurational-biased Monte Carlo (CBMC) simulations. High separation factors (6970) of H_2_O/CO_2_ could be achieved at 70 °C and 1.8 bar feed pressure. 

Three papers focused on the contribution of pore connectivity inside zeolite crystals to permeation performance. Kummali et al. [7] reported a molecular dynamics simulation to study the behavior of two fluids: ethane and CO_2_ confined in ZSM-22, a zeolite with channel-like pores of diameter 0.55 nm, which are isolated from each other. By comparing the behavior of the two fluids in ZSM-22 with that reported earlier in ZSM-5, and by artificially imposing pore connectivity in ZSM-22 by inserting a two-dimensional, slab-like, inter-crystalline space of thickness 0.5 nm, they studied the effect of the dimensionality and the geometry of pore connectivity. Consequently, while the translational motion of both ethane and CO_2_ in ZSM-22 is suppressed as a result of connecting the pores by perpendicular, quasi-one-dimensional pores of similar dimensions, the effect of connecting the pores by inserting the inter-crystalline space is different on the translational motion of the two fluids. Sakai et al. [8] investigated micropore volumes and effective pore sizes of silicalite-1 membranes by comparing them with those of a typical silicalite-1 powder. The silicalite-1 membrane with fewer grain boundaries showed similar micropore volume and effective pores size to those of the silicalite-1 powder, and exhibited relatively high permeation properties for C_6_-C_8_ hydrocarbons. In contrast, when the silicalite-1 membrane contained many grain boundaries, a relatively small micropore volume and effective pore size were observed, suggesting that the narrowing and obstruction of the micropore would occur along grain boundaries due to the disconnection of the zeolite pore. Sakai et al. concluded that it was important to reduce grain boundaries and improve pore connectivity to develop an effective preparation method for obtaining a highly permeable membrane. Sakai et al. [9] investigated the permeation behaviors of n-hexane and 2-methylpentane through two-types of silicalite-1 membranes that had different pore connectivity. The permeation mechanisms of these hydrocarbons were able to be explained by the adsorption–diffusion model, and the fluxes through silicalite-1 membranes could be expressed by the modified Fick’s first law. The hydrocarbon fluxes through the membrane with better pore connectivity were ca. 3–20 times larger than those through the membrane with poor pore connectivity. They concluded that the pore connectivity in the silicalite-1 membrane significantly influences molecular diffusivities. 

One paper explored the topic of the membrane reactor. Seshimo et al. [10] demonstrated the effect of a membrane reactor on methanol synthesis to improve one-pass CO_2_ conversion. They applied an Si-rich LTA membrane for dehydration from a methanol synthesis reaction field, and reported that the CO_2_ conversion of the membrane reactor was higher than that of the conventional packed-bed reactor under the all experimental conditions. This paper is a fascinating contribution as it demonstrates a new application of the LTA membrane.

This Special Issue aims to cover the recent developments and advances in all fundamental, application and industrial aspects that concern the microstructure and the separation performance of zeolite membranes. All of the articles published in this Special Issue were reviewed by recognized experts in the relevant fields of science. 
